# Clinical outcomes of cardiac arrest in pediatric patients presenting to the emergency department of a tertiary hospital in Kabul, Afghanistan: a retrospective cohort study

**DOI:** 10.1186/s12245-026-01180-7

**Published:** 2026-03-12

**Authors:** Mujeebullah Mahboob, Shafiqullah Shahim, Nijatullah Safi

**Affiliations:** 1 Department of Pediatrics, Emergency Medicine, French Medical Institute for Mothers and Children (FMIC), Kabul, Afghanistan; 2 Faculty of Public Health, Kabul University of Medical Sciences Abu Ali Ibn- e-Sina, Kabul, Afghanistan; 3 Department of Pediatrics Medicine, French Medical Institute for Mothers and Children (FMIC), Kabul, Afghanistan

**Keywords:** Pediatric, Cardiac arrest, Cardiopulmonary resuscitation, Clinical outcomes, Factors of survival, Emergency department, FMIC, Kabul, Afghanistan

## Abstract

**Introduction:**

Outcomes of pediatric cardiac arrest in our country remain suboptimal. Understanding the factors that influence these outcomes is essential for improving survival rates. This study aimed to evaluate the clinical outcomes of pediatric cardiac arrest in the emergency department and to identify the factors associated with these outcomes.

**Methods:**

A retrospective cohort study was conducted involving patients aged < 18 years who underwent cardiopulmonary resuscitation (CPR) in the emergency department of the French Medical Institute for Mothers and Children (FMIC) in Kabul, Afghanistan, between January 2021 and January 2025. Data were collected using the Utstein style of reporting. Bivariate and multivariable logistic regression analyses were performed to identify factors associated with survival outcomes.

**Results:**

Of the 200 patients who underwent CPR in the ED, sustained return of spontaneous circulation (ROSC) and survival to hospital discharge (STD) were achieved in 134 (67%) and 60 (30%) patients, respectively. Favorable neurological outcomes at hospital discharge were observed in 29 (14.5%) patients. Independent predictors of ROSC included IV/IO access established prior to arrest (AOR: 3.26, 95% CI: 1.68–6.3, *p* < 0.001), monitoring at the time of arrest (*p* = 0.003), administration of epinephrine during CPR (AOR: 18.3, 95% CI: 6.8–49.6, *p* < 0.001), endotracheal intubation during CPR (*p* < 0.001), and CPR duration ≤ 20 min (*p* < 0.001). Factors associated with survival to hospital discharge included response time ≤ 1 min (AOR: 7.2, 95% CI: 1.9–26.4, *p* = 0.003), time to first epinephrine dose ≤ 2 min (AOR: 5.3, 95% CI: 1.1–24.6, *p* = 0.03), administration of ≤ 2 doses of epinephrine during CPR (AOR: 5.3, 95% CI: 1.1–24.6, *p* = 0.03), CPR duration ≤ 15 min (*p* = 0.003), and post-resuscitation vasopressor/inotrope therapy (AOR: 5.79, 95% CI: 2.6–12.6, *p* < 0.001).

**Conclusion:**

Factors associated with favorable post-cardiac arrest outcomes included monitoring at the time of arrest, response time ≤ 1 min, endotracheal intubation during CPR, administration of ≤ 2 doses of epinephrine during CPR, and CPR duration ≤ 15 min. Early recognition, immediate cardiac monitoring for at-risk patients, and timely high-quality CPR may significantly improve post-arrest outcomes.

## Background

Cardiac arrest is a critical medical emergency defined as the sudden cessation of cardiac mechanical function, leading to inadequate circulation of blood. Clinically, this is evidenced by the absence of a palpable central pulse, apnea, loss of blood pressure, and unresponsiveness [1]. Pediatric cardiac arrest is relatively rare compared to cardiac arrest in adults; available data suggest that over 20,000 children undergo CPR annually in the United States alone. Of these, approximately 15,000 cases occur in-hospital and 7,000 out-of-hospital [2–6]. The true incidence of pediatric cardiac arrest in the emergency department is difficult to estimate due to inconsistencies in reporting, but it is generally less frequent than in adults, with a higher incidence in infants and decreasing rates with increasing age. The best data suggest incidence rates ranging from 2.6 to 19.7 annual cases per 100,000 pediatric population [2, 6–11]. According to a report from the American Heart Association’s National Registry of Cardiopulmonary Resuscitation (NRCPR), out of 23,535 in hospital cardiac arrests recorded over a 2-year period, only 778 (3,3%) occurred in children under 18 years of age. Of those, only 60 (8.9%) occurred in the pediatric emergency department [9, 12].

Unlike adults, pediatric cardiac arrest is less often the result of a primary cardiac event and is most commonly caused by progressive respiratory failure or shock. In these cases, cardiac arrest is typically preceded by a period of physiological deterioration, eventually leading to cardiopulmonary failure, bradycardia, and cardiac arrest. In contrast, children with congenital heart diseases (CHDs) and those who have undergone cardiac surgery often experience cardiac arrest due to a primary cardiac cause [2, 6, 13].

Pediatric cardiac arrest is an intervenable process for which outcomes have significantly improved over the past two decades, largely due to advancements in pre-hospital emergency care, including the provision of community-based bystander CPR, delivery of high-quality in-hospital CPR, and adequate post-resuscitation care [2, 5, 14]. According to data from the American Heart Association’s Get With The Guidelines-Resuscitation Registry (GWTG-R), approximately 80% of pediatric in-hospital cardiac arrests achieve sustained return of spontaneous circulation (ROSC) for more than 20 min, with 20–40% surviving to hospital discharge, and nearly 47% of those survivors having favorable neurologic outcomes [2, 5, 15, 16]. However, limited data exist regarding the clinical characteristics and outcomes of pediatric cardiac arrest specifically in the emergency department. The available evidence suggests that worldwide survival rates for pediatric cardiac arrest in the emergency department range between 12.8% and 33.8% [3].

Critical factors influencing survival outcomes in pediatric cardiac arrest include the environment where the arrest occurs (in-hospital vs. out-of hospital), mechanism of arrest (traumatic vs. non-traumatic), pre-existing medical conditions, whether the event was witnessed, patient monitoring at the time of arrest, initial electrocardiographic rhythm, no-flow time duration (the period without spontaneous circulation or CPR), total duration of CPR, total dosage of epinephrine administered, and the quality of life-support therapies provided both during and after resuscitation, particularly the provision of bystander CPR in pre-hospital settings [7, 10, 17–19]. Emerging evidence suggests that appropriate, time-sensitive interventions applied during four distinct phases of cardiac arrest can improve survival and neurologic outcome in children: [1] pre-arrest phase (protect) [2], arrest or no-flow phase (preserve) [3], low-flow or CPR phase (resuscitate), and [4] post-resuscitation phase (regenerate). These interventions include early identification of at-risk patients, prompt defibrillation for shockable rhythms, delivery of high-quality CPR, and goal-directed post-resuscitation care [6, 7, 10].

Globally, data on the clinical characteristics, causes, and outcomes of pediatric cardiac arrest in the emergency department setting is limited. Most available studies originate from high-income countries (HICs), whose findings may not be directly applicable to low- and middle-income countries (LMICs) due to differing disease profiles (e.g., higher prevalence of sepsis and infectious diseases), delayed access to care, and resource-constrained healthcare systems. Additionally, the higher incidence of cardiac arrest in LMICs suggests that both the outcomes and the factors influencing these outcomes differ from those in HICs, making extrapolation of data from high-income settings unreliable for low- and middle-income contexts [1, 3, 20–22].

In Afghanistan, as in many other developing countries, unique healthcare challenges hinder the systematic tracking and documentation of cardiac arrest outcomes in hospital settings. The country faces fragile healthcare infrastructure, inconsistent availability and quality of healthcare services, and significant sociopolitical and cultural barriers to accessing timely, high-quality care. Specific challenges contributing to poor outcomes following cardiac arrest include inadequate medical record systems, a lack of basic life support (BLS) training for healthcare personnel and general public, insufficient pre-hospital ambulance and paramedical services even in urban areas, prolonged emergency response times, and the frequent transport of cardiac arrest patients to hospitals in private vehicles by untrained individuals, receiving little to no life support during the critical pre-hospital phase. Moreover, the quality and effectiveness of CPR provided in hospital settings is often suboptimal [23, 24].

To date, no studies have addressed the clinical characteristics and outcomes of pediatric cardiac arrest in emergency department settings in Afghanistan. Therefore, this is the first study using the Utstein-style reporting guidelines to evaluate the clinical outcomes of pediatric cardiac arrest and identify factors influencing these outcomes in the emergency department setting of the French Medical Institute for Mothers and Children (FMIC) in Kabul, Afghanistan.

## Methods

### The aim, design and setting of the study

This study aimed to evaluate the clinical outcomes of pediatric in-hospital cardiac arrest and identify factors influencing these outcomes in the Emergency Department (ED) of the French Medical Institute for Mothers and Children (FMIC) in Kabul, Afghanistan. It was designed as a retrospective cohort study of all pediatric patients who underwent CPR in the emergency department of FMIC between January 2021 and January 2025.

The study was conducted at the ED of FMIC, a 176-bed tertiary referral and teaching hospital in Kabul, Afghanistan. Since its founding in 2006, FMIC has operated as a not-for-profit private hospital through a unique collaboration among the Afghan and French governments, the Aga Khan Development Network, and the NGO La Chaîne de l’Espoir. Despite its name, FMIC serves as a general hospital, providing high-quality healthcare services to all age groups, including newborns, children, adolescents, and adults.

The emergency department of FMIC includes 10 monitored beds and 2 resuscitation beds, providing emergency care to around 100 patients daily, of whom nearly two quarter are pediatric patients of less than 18 years of age. Staffed by emergency physicians and nurses trained in Basic Life Support (BLS), Pediatric Advanced Life Support (PALS), and Neonatal Resuscitation Program (NRP), the ED offers specialized pediatric and neonatal emergency services vital for this study’s focus on pediatric cardiac arrest outcomes.

The ED features a dedicated resuscitation room with advanced resources, including a crash cart with essential equipment and medications, a ventilator, and continuous monitoring systems in accordance with AHA guidelines. FMIC also maintains a separate Code Blue team comprising a pediatric emergency physician, an intensivist, nurses, and an on-call anesthesiologist, who respond to rush call and provide CPR when needed.

### Study participants

The study included all patients under 18 years of age who underwent cardiopulmonary resuscitation (CPR) in the emergency department of FMIC between January 2021 and January 2025. This included all cases who received CPR for pulseless cardiac arrests, non-pulseless cardiac arrests (bradycardia with poor perfusion), and respiratory arrests who received assisted ventilation and subsequently required CPR. Outcomes were analyzed as a single cohort without stratifying by arrest subtype. Patients for whom resuscitation was not attempted, including cases with pronounced dead-on arrival to the ED, those with a Do Not Resuscitate (DNR) order, and cases with incomplete records (lack of a CPR sheet containing key resuscitation variables such as response time, CPR duration, number of epinephrine doses administered, initial cardiac rhythm, and the outcome variables) were excluded.

### Data collection

Data were collected using a standardized and pretested data collection form, designed based on Pediatric Utstein-style reporting guidelines. The Pediatric Utstein style of reporting provides a globally recognized standard for pediatric resuscitation research and focus on the consistent reporting of cardiac arrest and cardiopulmonary resuscitation outcomes [25]. This standardized approach facilitates uniform data collection, enhancing the comparability of our findings with international data and strengthening the study’s validity.

In the emergency department of FMIC, patients who presented with cardiac arrest were labeled as Code Blue cases and recorded in the emergency department resuscitation room logbook based on their medical record numbers. These medical record numbers were then used to locate and retrieve the patients’ files from the medical records department. After a thorough review of the medical records, including the CPR sheet, data were documented across five categories of variables: patient variables, arrest variables, resuscitation-related variables, post-resuscitation variables, and outcome variables.

### Study variables

The primary outcome variables were sustained return of spontaneous circulation (ROSC) and survival to hospital discharge (STD). Secondary outcome variables included survival to hospital admission (STA) and neurological status at hospital discharge. These outcomes were determined based on the clinical information documented in the patients’ files, including the CPR sheets contained within them.

ROSC was defined as the restoration of palpable, spontaneous central pulses maintained for more than 20 min in a cardiac arrest patient. Survival to discharge (STD) was defined as patient survival with ROSC, followed by discharge from the hospital alive or transfer to another healthcare facility after a minimum stay of 24 h post-ROSC. Survival to hospital admission (STA) defined as patient survival with ROSC resulting in transfer from the emergency department to the ICU or subsequently from the ICU to a general pediatric ward.

To evaluate the cognitive function and neurological status (cerebral capabilities) of patients following cardiac arrest at the time of hospital discharge the Pediatric Cerebral Performance Category (PCPC) Scale was used. Scores of 1 or 2 were considered good or favorable neurological outcomes, indicating either normal function or mild to moderate impairment. Scores of 3 or higher were categorized as poor or unfavorable outcomes, indicating severe disability, vegetative state, or brain death.

Independent variables included (1) patient variables such as patient demographics, referral status, pre-arrest vital signs, pre-existing medical conditions, provisional diagnoses, and treatments and interventions given prior to arrest; (2) arrest variables such as the immediate cause of arrest, and initial documented cardiac rhythm; (3) resuscitation-related variables such as chest compressions, defibrillation, endotracheal intubation during CPR, use of vasoactive drugs during CPR, response time, CPR duration, number of epinephrine doses administered during CPR, and time to first epinephrine dose; and (4) post-resuscitation variables such as targeted temperature management (TTM), mechanical ventilation, and vasopressor or inotrope therapy following resuscitation.

Provisional diagnoses were abstracted from chart documentation and categorized as independent variables. Some diagnoses, particularly sepsis, may contribute to multiple downstream pathways leading to cardiac arrest. In such cases, the diagnosis was classified according to the primary etiology documented by the treating team. Weight was categorized operationally (≤ 5, 6–10, 11–20, > 20 kg) to facilitate data presentation and interpretation. These categories were not based on clinical or practical cut points.

Monitoring at the time of arrest was defined as patients attached to a bedside monitor and observed by a healthcare professional (physician, nurse, or other staff). The initial ECG rhythm was documented only when it was clearly recorded in the medical records.

### Statistical analysis

Statistical analysis was conducted in four stages. (1) Data were entered and managed using IBM SPSS Statistics for Windows, Version 25.0 (IBM Corp., Armonk, NY, USA). (2) Data cleaning was performed by generating frequency distributions for all variables to identify out-of-range values, missing data, and insufficient variability. Independent variables with dichotomous (yes/no) response categories that had five or fewer missing observations were recoded as “No” for the purpose of analysis (e.g., congenital heart disease, sepsis, lower respiratory tract infection, respiratory arrest, shock, intravenous crystalloid fluids, vasopressors or inotropes, and intravenous or intraosseous access). (3) Descriptive statistics were used to describe patients’ demographics, clinical status, and resuscitation details. For categorical variables (e.g., gender, referral status), frequencies and percentages were reported. For continuous variables (e.g., weight, CPR duration), measures of central tendency and variability, such as mean, median, standard deviation, and interquartile range (IQR), were calculated. (4) Chi-square tests were used to explore associations between categorical variables. Binary logistic regression was performed to examine the association between the outcome variable and each independent variable, and unadjusted odds ratios (UORs) with 95% confidence intervals (CIs) were calculated. Independent variables with *p* < 0.05 in bivariate analysis were entered into the multivariable logistic regression model to estimate adjusted odds ratios (AORs) with 95% confidence intervals. These variables included socio-demographic factors, sepsis, established IV/IO access prior to arrest, antibiotic therapy prior to arrest, vasopressors/inotropes therapy prior to arrest, monitoring at the time of arrest, initial rhythm, endotracheal intubation during CPR, number of epinephrine doses administered, response time, and CPR duration.

## Results

**Fig. 1:**
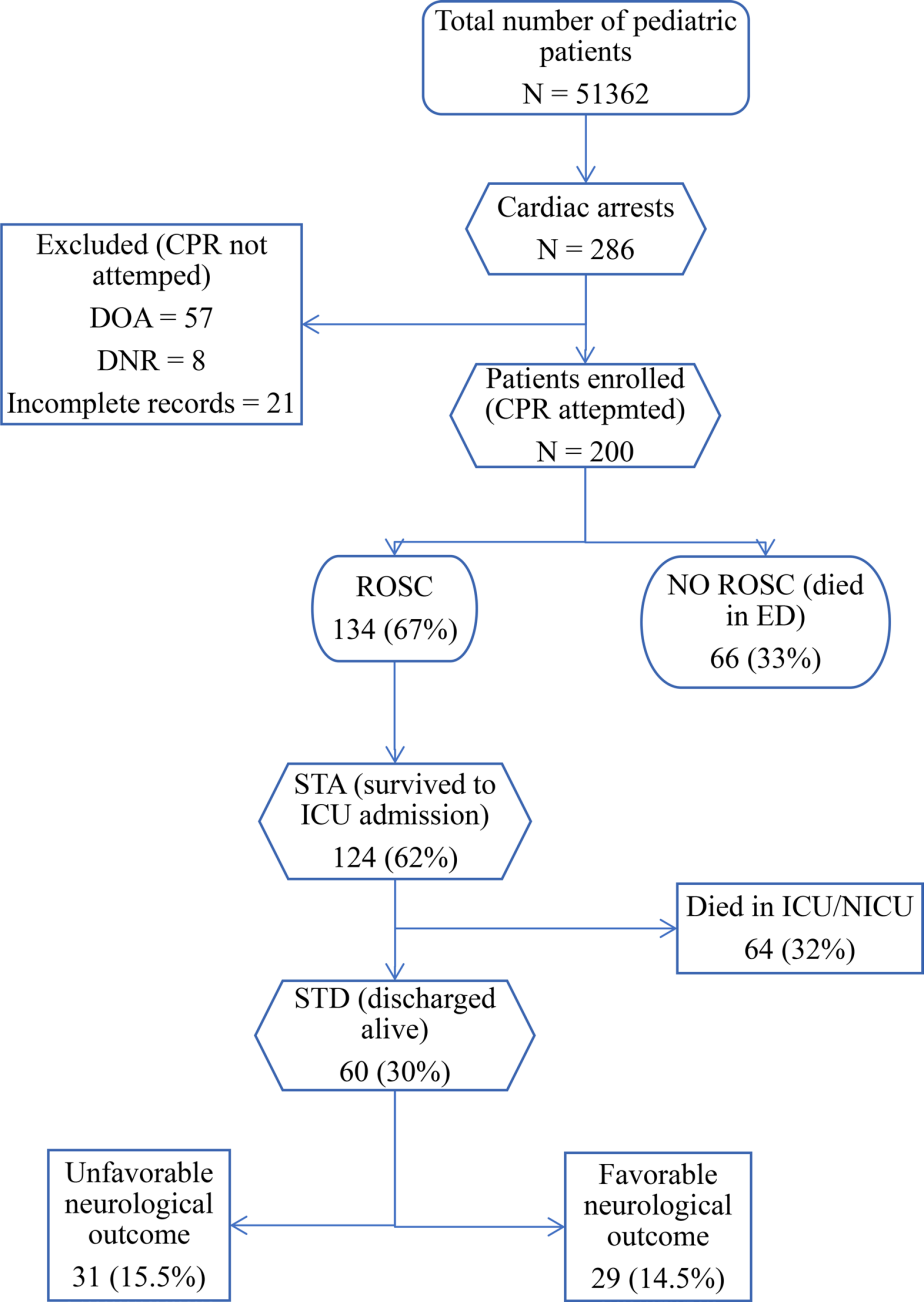
Patients’ inclusion flowchart and outcomes of cardiac arrest in the emergency department, FMIC, Kabul, Afghanistan. ROSC: Return of spontaneous circulation, STA: Survival to admission, STD: Survival to discharge, ED: Emergency department, ICU: intensive care unit, NICU: neonatal intensive care unit

A total of 51,362 pediatric patients were registered in the emergency department of FMIC from January 2021 to January 2025. Among these, 286 patients developed cardiac arrest and were screened based on the eligibility criteria for possible inclusion in the study. Eighty-six patients were excluded: 57 patients were labeled as Death on Arrival (DOA), 8 patients had signed a Do Not Resuscitate (DNR) order, and 21 patients had incomplete records. Ultimately, 200 patients who underwent CPR met the inclusion criteria of the study. The incidence of cardiac arrest was 5.6 per 1000 ED admissions (Fig. 1).

### Demographic and clinical characteristics of patients prior to cardiac arrest

Of the total participants, 141 (70.5%) were male, and 59 (29.5%) were female. Regarding age distribution, the majority (139, 69.5%) were infants under one year of age, followed by children aged 1–5 years. The median weight was 5.0 kg (IQR: 3.0–10.0 kg), with 90 participants (45.0%) weighing less than 5.0 kg. The majority of the study population (146, 73%) were referred from other healthcare facilities (Table 1).

**Fig. 2:**
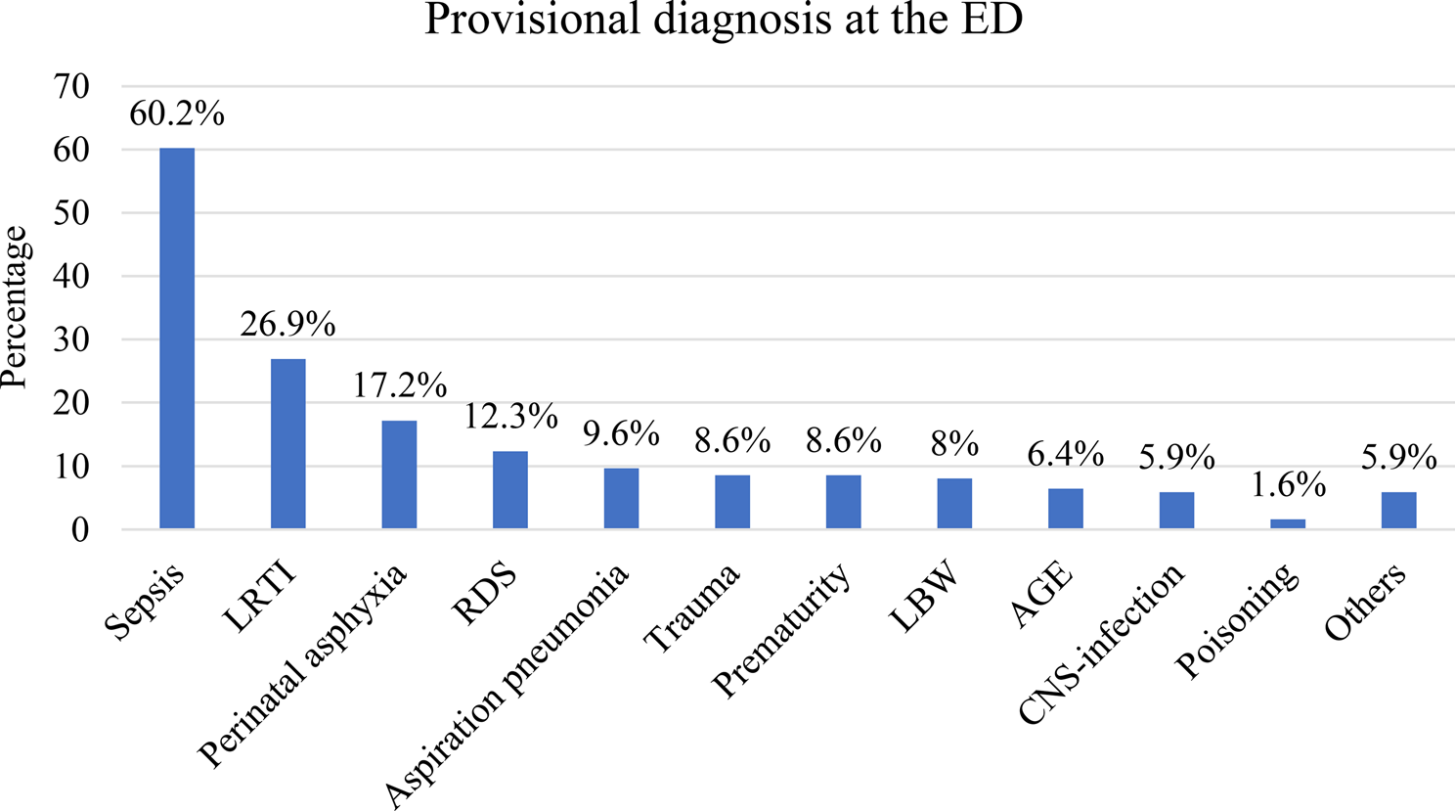
Provisional diagnoses of patients in the emergency department, FMIC, Kabul, Afghanistan. Others include bronchial foreign body, dilated cardiomyopathy (DCM), intracranial hemorrhage (ICH), malignancy, sudden infant death syndrome (SIDS), and blood transfusion reaction/anaphylaxis. AGE: Acute gastroenteritis, CNS: Central nervous system, LRTI: Lower respiratory tract infections, RDS: Respiratory distress syndrome. NB: One patient may have more than one diagnosis (multiple responses)

Overall, 64 (32%) of the patients had pre-existing diseases, with congenital heart diseases (51.6%) being the most commonly reported condition, followed by seizure disorder (23.4%). The most frequently reported provisional diagnosis in the ED was sepsis (112, 60.2%), followed by lower respiratory tract infections (50, 26.9%) (Fig. 2). During the initial assessment in ED triage, hypoxia 185 (95.4%) was the most frequently recorded abnormal vital sign, followed by hypotension 118 (60.8%) and bradycardia 81 (41.8%). In the pre-arrest phase, 74.9% of patients had established IV/IO access, and 11.6% were intubated. Oxygen therapy was administered to 65.3% of patients as a pre-arrest treatment, and 30.2% received intravenous crystalloid fluids prior to arrest (Table 1).

**Table 1:** Demographic and clinical characteristics of pediatric cardiac arrest patients presenting to the emergency department, FMIC, Kabul, Afghanistan (*n* = 200, 2025)

Variables	Median (IQR)	Frequency (*n*)	Percent (%) ^*^
**1. Patient variables**			
**Age groups**			
< 1 year		139	69.5^*^
1–5 years		31	15.5
6–12 years		18	9.0
13–18 years		12	6.0
**Gender**			
Male		141	70.5^*^
Female		59	29.5
**Weight groups**	5.0 kg (3.0–10.0)		
≤ 5 kg		90	45.0^*^
6 to 10 kg		44	22.0
11 to 20 kg		21	10.5
>20 kg		22	11.0
Unknown		23	11.5
**2. Arrest variables**			
**Referral Status (referred)**		146	73.0^*^
Pre-existing conditions		64	32.0^*^
CHD		33	51.6
Seizure disorder		15	23.4
Malnutrition		8	12.5
Down syndrome		5	7.8
CP		3	4.7
**Etiology (ED provisional diagnosis)** ^***^		186	93.0^*^
Sepsis		112	60.2
LRTI		50	26.9
Trauma		16	8.6
CNS infection		11	5.9
AGE		12	6.5
Poisoning		3	1.6
**Immediate cause of arrest**		190	95.0^*^
Respiratory arrest		131	68.9
Shock		72	37.9
Acidosis		45	23.7
Hyperkalemia		21	11.1
Hypokalemia		5	2.6
Arrhythmia		1	0.5
**Pre-arrest vital signs**		194	97.0^*^
Hypoxia		185	95.4
Hypotension		118	60.8
Bradycardia		81	41.8
Tachycardia		40	20.6
Fever		22	11.3
Pre-arrest Treatment		199	99.5^*^
Oxygen therapy		130	65.3
IV crystalloid fluids		60	30.2
Vasopressors, inotropes		15	7.5
Antibiotic therapy		9	4.5
Pre-arrest Interventions		151	75.5^*^
IV/IO access		149	74.9
Endotracheal intubation		23	11.6
Arterial line		1	0.5
**3. Resuscitation-related variables**			
**Monitored at the time of arrest** ^**^		113	56.5^*^
**Initial rhythm** ^**^		83	41.5^*^
Asystole		71	35.5
VF/pVT		4	2.0
PEA		7	3.5
SVT		1	0.5
**Chest compression**		194	97.0^*^
**Airway management**			
Bag-valve-mask		188	94.0^*^
Endotracheal tube		130	65.0
Tracheostomy		1	0.5
**Defibrillation**		2	1.0
**Medications given during CPR**		119	59.5^*^
Epinephrine		117	58.5
Calcium		55	27.5
Sodium bicarbonate		41	20.5
Atropine		11	5.5
Amiodarone		3	1.5
Lidocaine		3	1.5
**Epinephrine doses**	3 doses (2–4)		
**Time to first epinephrine (min)**	3 min (1–5)		
**Response time (min)**	1 min (1–1)		
**CPR duration (min)**	10 min (5–20)		
**4. Post-resuscitation variables**			
Mechanical ventilation		126	63.0^*^
Vasopressors or inotropes		76	38.0

^*^ Percentages are calculated based on the total study population (denominators = 200)

^**^ “Monitored” refers to patients attached to a bedside monitor and observed by healthcare personnel; initial rhythm recorded only if documented

^***^ NB: One patient may have more than one diagnosis (multiple responses), and the small percentages are not reported here.AGE: acute gastroenteritis, CHD: congenital heart diseases, CNS: central nervous system, CP: cerebral palsy, CPR: cardiopulmonary resuscitation, ED: emergency department, IV/IO: intravenous/intraosseous, IQR: interquartile range, LBW: low birth weight, LRTI: lower respiratory tract infections, PEA: pulseless electrical activity, pVT: pulseless ventricular tachycardia, RDS: respiratory distress syndrome, SVT: supraventricular tachycardia, VF: ventricular fibrillation, VT: ventricular tachycardia

### Characteristics of cardiac arrest event and cardiopulmonary resuscitation

In 95% of cases, the immediate physiological deterioration leading to cardiac arrest was identified. The most commonly reported precipitating event was respiratory arrest (68.9%), followed by shock (37.9%). Overall, 56.5% of patients who experienced cardiac arrest were being monitored at the time of the event. The initial cardiac rhythm was documented in 41.5% of cases, with non-shockable rhythms identified in 39.5% (asystole 35.5%, PEA 3.5%). Only 2% of cases had shockable rhythms (VF/pVT) (Table 1).

In this cohort, 91% of patients underwent chest compressions with bag-mask ventilation, while 62% received chest compressions with advanced airway management through endotracheal intubation. During CPR, 119 (59.5%) patients received resuscitation drugs, with epinephrine administered to 58.5% of the patients. The median number of epinephrine doses administered was 3 (IQR: 2–4). Defibrillation was performed in two patients. The median time to first epinephrine dose was 3 min (IQR: 1–5 min), the median response time was 1 min (IQR: 1–1 min), and the median duration of CPR was 10 min (IQR: 5–20 min) overall. In the post-resuscitation phase, 63.0% of patients were put on mechanical ventilation, and 38.0% received vasopressors/inotropes. None of the study participants underwent targeted temperature management (TTM) during this phase (Table 1).

### Outcomes of pediatric cardiac arrest in the emergency department

Out of 200 patients who experienced cardiac arrest and underwent CPR in the ED, 134 (67%) attained ROSC for more than 20 min, and 124 (62%) survived to ICU admission. Of these, 64 (32%) died in the ICU, and 60 (30%) survived to hospital discharge. Among patients who discharged alive, 31 (15.5%) had unfavorable neurological outcomes, while 29 (14.5%) had favorable neurological outcomes, as assessed by the pediatric cerebral performance category (PCPC). Overall, 66 (33%) patients died in the emergency department (Fig. 1).

### Bivariate and multivariable logistic regression analysis of factors associated with return of spontaneous circulation (ROSC)

Table 2 presents the factors associated with return of spontaneous circulation (ROSC) in the emergency department. In the pre-arrest phase, the following factors were significantly associated with ROSC based on bivariate and univariate analyses: the 1–5 years age group, gender, sepsis, antibiotic therapy, oxygen therapy, and IV/IO access in place prior to cardiac arrest. In the multivariable analysis (Table 2) after controlling for confounders, factors that were independently associated with ROSC were 1–5 years age group, sepsis, oxygen therapy, and IV/IO access in place prior to cardiac arrest. The 1–5 years age group was associated with 2.2 times higher odds of achieving ROSC (AOR: 2.2, 95% CI: 1.002–5.2, *p* = 0.04, reference: <1 year). Having IV/IO access prior to cardiac arrest was associated with 3.26 times higher odds of achieving ROSC (AOR: 3.26, 95% CI: 1.68–6.3, *p* < 0.001) compared to patients without IV/IO access before arrest.

In the resuscitation phase, factors significantly associated with ROSC based on bivariate and univariate analyses included monitoring at the time of arrest, a shockable initial cardiac rhythm, endotracheal intubation, administration of ≤ 2 doses of epinephrine, and CPR duration ≤ 20 min. In the multivariable analysis, monitoring at the time of arrest, endotracheal intubation, administration of ≤ 2 doses of epinephrine, and CPR duration ≤ 20 min remained significantly associated with ROSC. The administration of epinephrine during resuscitation was a strong predictor of ROSC, associated with 18.3 times higher odds of achieving ROSC (AOR: 18.3, 95% CI: 6.8–49.6, *p* < 0.001) (Table 2).

**Table 2:** Bivariate and multivariable logistic regression analysis of factors associated with return of spontaneous circulation (ROSC) following pediatric cardiac arrest in the emergency department, FMIC, Kabul, Afghanistan (*n* = 134, 2025)

Variables	ROSC achieved^1^			
	UOR (95%CI)	*p* -value	AOR (95%CI)	*p* -value
**Age groups**				
< 1 year	Ref	0.14^**^	Ref	0.16
1–5 years	2.4 (1.08–5.3)	**0.03** ^**^	2.2 (1.002 -5.2)	**0.04**
6–12 years	1.6 (0.5–4.5)	0.34^**^	1.3 (0.4–3.8)	0.59
13–18 years	1.8 (0.5–6.1)	0.32^**^	2.3 (0.6–8.3)	0.17
**Gender**				
Male	1.9 (1.05–3.7)	**0.03** ^*^	0.6 (0.2–1.2)	0.18
Female	Ref		Ref	
**Sepsis**				
Yes	2.28 (1.2–4.1)	**0.007** ^*^	0.49 (0.2–1)	**0.02**
No	Ref		Ref	
**Antibiotic therapy**				
Yes	1.5 (1.4–1.7)	**0.03** ^*^	N/A	0.999
No	Ref			
**Oxygen therapy**				
Yes	2.4 (1.3–4.38)	**0.005** ^*^	0.42 (0.2–0.8)	**0.007**
No	Ref		Ref	
**IV/IO access**				
Yes	3.5 (1.8–6.9)	**< 0.001** ^*^	3.26 (1.68–6.3)	**< 0.001**
No	Ref		Ref	
**Monitored at the time of arrest**				
Yes	2.9 (1.4–4.73)	**0.002** ^*^	0.4 (0.2–0.7)	**0.003**
No	Ref		Ref	
**Initial rhythm**				
Shockable	2.13 (1.7–2.7)	**0.03** ^*^	N/A	0.99
Non-shockable	Ref			
**Endotracheal intubation**				
Yes	8.3 (4.2–16.1)	**< 0.001** ^*^	0.17 (0.05–0.21)	**< 0.001**
No	Ref		Ref	
**Epinephrine doses**				
≤ 2 doses	4.9 (2.1–11.06)	**< 0.001** ^*^	0.18 (0.07–0.4)	**< 0.001**
> 2 doses	Ref		Ref	
**CPR duration (min)**				
≤ 20 min	20.5 (8.3–50.4)	**< 0.001** ^*^	0.04 (0.01–0.1)	**< 0.001**
> 20 min	Ref		Ref	

^1^ROSC not achieved is the reference category, ^*^Chi-square/mann-whitney u tests, ^**^univariate binary logistic regression, ^***^multivariable binary logistic regression (backward method). UOR: unadjusted odds ratio, AOR: adjusted odds ratio, IQR: interquartile range, Ref: reference category, N/A: not applicable, CHD: congenital heart diseases, CP: cerebral palsy, ED: emergency department, LRTI: lower respiratory tract infections, CNS: central nervous system, AGE: acute gastroenteritis, RDS: respiratory distress syndrome, LBW: low birth weight, CPR: cardiopulmonary resuscitation, IV/IO: intravenous/intraosseous, VF: ventricular fibrillation, VT: ventricular tachycardia, SVT: supraventricular tachycardia, PEA: pulseless electrical activity, pVT: pulseless ventricular tachycardia

### Bivariate and multivariable logistic regression analysis of factors associated with survival to discharge (STD)

Table 3 presents the factors associated with survival to discharge (STD). In bivariate and univariate analyses, 7 variables were significantly associated with survival to discharge including sepsis, vasopressor/inotrope use prior to arrest, administration of ≤ 2 doses of epinephrine, time to first epinephrine dose ≤ 2 min, response time ≤ 1 min, CPR duration ≤ 15 min, and post-resuscitation vasopressor/inotrope use.

Multivariable analysis (Table 3) identified sepsis, administration of ≤ 2 doses of epinephrine during CPR, time to first epinephrine dose ≤ 2 min, response time ≤ 1 min, CPR duration ≤ 15 min, and post-resuscitation vasopressor/inotrope use as independent predictors of STD.

Administration of the first dose of epinephrine within ≤ 2 min of arrest recognition was associated with 5.3-fold higher odds of STD (AOR: 5.3, 95% CI: 1.1–24.6, *p* = 0.03). A response time of ≤ 1 min was associated with 7.2 times greater odds of STD (AOR: 7.2, 95% CI: 1.9–26.4, *p* = 0.003). Post-resuscitation vasopressor/inotrope use was associated with 5.79-fold higher odds of STD (AOR: 5.79, 95% CI: 2.6–12.6, *p* < 0.001) (Table 3).

**Table 3:** Bivariate and multivariable logistic regression analysis of factors associated with survival to discharge (STD) following pediatric cardiac arrest in the emergency department, FMIC, Kabul, Afghanistan (*n* = 60, 2025)

Variables	Survival to hospital discharge (STD)^1^			
	UOR (95%CI)	*p* - value	AOR (95%CI)	*p* - value
Sepsis				
Yes	0.4 (0.2–0.98)	**0.04** ^*^	2.06 (1.01–4.2)	**0.04**
No	Ref		Ref	
Vasopressors/inotropes (prior arrest				
Yes	1.9 (1.6–2.3)	**0.002** ^*^	−	0.999
No	Ref			
Epinephrine doses administered during CPR				
≤ 2 doses	7.52 (1.5–37.6)	**0.007** ^*^	0.1 (0.02–0.76)	**0.02**
> 2 doses	Ref		Ref	
Time to first epinephrine dose (min)				
≤ 2 min	0.2 (0.07–0.9)	**0.03** ^*^	5.3 (1.1–24.6)	**0.03**
> 2 min	Ref		Ref	
Response time (min)				
≤ 1 min	0.13 (0.03–0.5)	**0.001**	7.2 (1.9–26.4)	**0.003**
> 1 min	Ref		Ref	
CPR duration (min)				
≤ 15 min	10.0 (2.2–45.0)	**< 0.001** ^*^	0.1 (0.02–0.4)	**0.003**
> 15 min	Ref		Ref	
Vasopressors/inotropes (post-resuscitation)				
Yes	0.19 (0.09–0.4)	**< 0.001** ^*^	5.79 (2.6–12.6)	**< 0.001**
No	Ref		Ref	

^1^Did not survive to hospital discharge is the reference category, ^*^Chi-square/mann-whitney u tests, ^**^univariate binary logistic regression, ^***^multivariable binary logistic regression (backward method). UOR: unadjusted odds ratio, AOR: adjusted odds ratio, Ref: reference category, SD: standard deviation, CPR: cardiopulmonary resuscitation

### Bivariate and multivariable logistic regression analysis of factors associated with neurological outcomes at hospital discharge

Table 4 presents the factors associated with favorable neurological outcomes at hospital discharge. In bivariate and univariate analyses, 5 variables were significantly associated with a favorable neurological prognosis, including mechanical ventilation prior to arrest, endotracheal intubation during CPR, a response time ≤ 1 min, CPR duration ≤ 5 min, and post-resuscitation vasopressor/inotrope use. In multivariable analysis (Table 4), 5 variables remained significantly associated with favorable neurological outcomes, including mechanical ventilation prior to arrest, endotracheal intubation during CPR, response time ≤ 1 min, CPR duration ≤ 5 min, and post-resuscitation vasopressor/inotrope use. Endotracheal intubation during CPR was associated with 4.9 times greater odds of a favorable neurological outcome (AOR: 4.9, 95% CI: 1.1–20.2, *p* = 0.02). Post-resuscitation vasopressor or inotrope use was associated with 3.5 times greater odds of a favorable neurological outcome (AOR: 3.5, 95% CI: 1.02–12.3, *p* = 0.04) (Table 4).

**Table 4:** Bivariate and multivariable logistic regression analysis of factors associated with neurological outcomes at hospital discharge following pediatric cardiac arrest in the emergency department, FMIC, Kabul, Afghanistan (*n* = 29, 2025)

Variables	Favorable neurological outcome at hospital discharge^1^			
	UOR (95%CI)	*p* - value	AOR (95%CI)	*p* - value
Mechanical ventilation (prior cardiac arrest)				
Yes	0.14 (0.01–1.3)	**0.05** ^*^	10.6(1.05–108.1)	**0.04**
No	Ref		Ref	
Endotracheal intubation (during CPR)				
Yes	0.2 (0.04–0.83)	**0.02** ^*^	4.9 (1.1–20.2)	**0.02**
No	Ref		Ref	
Response time (min)				
≤ 1 min	4.76 (1.1–19.3)	**0.02** ^*^	0.17 (0.03–0.9)	**0.04**
> 1 min	Ref		Ref	
CPR duration (min)				
≤ 5 min	4.04 (1.3–11.8)	**0.01** ^*^	0.24 (0.08–0.7)	**0.01**
> 5 min	Ref		Ref	
Vasopressors or inotropes (post-resuscitation)				
Yes	0.2 (0.08–0.87)	**0.02** ^*^	3.5 (1.02–12.3)	**0.04**
No	Ref		Ref	

^1^Unfavorable neurological outcome at hospital discharge is the reference category, ^*^Chi-square/mann-whitney u tests, ^**^univariate binary logistic regression, ^***^multivariable binary logistic regression (backward method). UOR: unadjusted odds ratio, AOR: adjusted odds ratio, Ref: reference category, IQR: interquartile range, SD: standard deviation, CPR: cardiopulmonary resuscitation

## Discussion

The present study is one of the first to apply the Utstein reporting guidelines to evaluate the clinical outcomes of cardiac arrest and to identify factors influencing these outcomes in pediatric patients presenting to the emergency department of a tertiary-level hospital in Kabul, Afghanistan. We systematically analyzed the association between multiple pre-arrest, arrest, and post-arrest factors with four key outcomes of cardiac arrest: return of spontaneous circulation (ROSC), survival to admission (STA), survival to discharge (STD), and neurological outcomes at hospital discharge. Several factors were found to be independently associated with each of these outcomes.

We found that two-thirds (67%) of patients attained sustained ROSC in the emergency department, and almost one-third (30%) were discharged alive from the hospital. Although data on pediatric cardiac arrest in emergency departments (EDs) is limited, Michelson et al., in a retrospective study using the 2009–2014 Nationwide Emergency Department Sample (NEDS), reported a survival to discharge (STD) rate of 33.8% for non-traumatic cardiac arrests in pediatric emergency department, which closely aligns with our findings [11].

Studies from high-income countries have reported higher survival rates than those observed in our study [5, 18, 26]. Girotra et al., using data from the Get With The Guidelines–Resuscitation (GWTG-R) registry, reported a ROSC rate of 81.2% and a STD rate of 43.4% for in-hospital cardiac arrest (IHCA), both notably higher than our findings. The differences may be attributed to several factors, including the quality of pre-hospital care, the availability of bystander CPR, and the adequacy of post-resuscitation care. Pediatric cardiac arrest outcomes depend not only on the delivery of high-quality CPR within the hospital but also on effective pre-hospital emergency care and comprehensive post-resuscitation management [6, 10, 17].

In contrast, studies from low and middle income countries and low resource settings report generally lower survival rates and poorer neurological outcomes following pediatric cardiac arrest compared with high income countries [21, 22, 27, 28]. Mally et al., in a retrospective ED-based study from Tanzania, reported a ROSC rate of 51.5% and an STD rate of just 5.2% for pediatric cardiac arrest, both significantly lower than our findings. Similarly, Alsabri et al., in a narrative review of low-resource pediatric emergency departments, highlighted survival rates often below 20%, frequently accompanied by substantial neurological impairment, reflecting challenges related to delayed presentation, limited infrastructure, and constrained post-arrest care in these settings. The differences between our results and those from other low-resource settings may partly be explained by the fact that our study adopted broad inclusion criteria encompassing all events in which ventilation or chest compressions were initiated, including pulseless cardiac arrests, non-pulseless cardiac arrests (bradycardia with poor perfusion), and respiratory arrests.

Another contributing factor to our relatively favorable outcomes could be the setting of our study. It was conducted in a tertiary hospital equipped with a well-equipped emergency department staffed by emergency physicians, medical officers, and nurses trained in Basic Life Support (BLS), Pediatric Advanced Life Support (PALS), and the Neonatal Resuscitation Program (NRP). Our ED features a dedicated resuscitation room with advanced resources, including a crash cart with essential equipment and medications, a ventilator, and continuous monitoring systems in accordance with AHA guidelines. FMIC has a separate code blue team comprising a pediatric emergency physician, an intensivist, nurses, and an on-call anesthesiologist who respond to rush call and provide CPR. Studies have shown that hospitals with well-trained personnel and structured resuscitation protocols achieve better survival outcomes in cardiac arrest cases [19].

In our study, 14.5% of patients had favorable neurological outcomes at hospital discharge, as assessed by the Pediatric Cerebral Performance Category (PCPC). Comparatively, Shimoda-Sakano et al., reported that 76.1% of pediatric IHCA survivors had favorable neurological outcomes at hospital discharge, and Rathore et al., found that three-quarters of survivors had favorable outcomes at one-year follow-up [18, 20]. In contrast, Lee et al., reported that only 4.6% of pediatric cardiac arrest survivors were discharged with favorable neurological outcomes [29]. One possible explanation for the differences between our findings and those of international studies is the lack of data on the baseline or pre-arrest neurological status of patients in our cohort. Our findings indicate that the majority (73%) of study participants were referred from other health facilities, where a major challenge was the unavailability of data on their pre-arrest neurological status, initial presentation, and pre-hospital treatment. The high referral rate may also have contributed to prolonged no-flow (time from arrest to initiation of CPR) or low-flow (duration of CPR prior to return of spontaneous circulation) times before arrival, which negatively impacts brain recovery. Furthermore, due to the retrospective nature of this study, we were unable to assess the quality of CPR administered, which may have impacted survival outcomes.

Assessing neurological outcomes following pediatric cardiac arrest remains challenging due to variability in reporting metrics and follow-up durations; however, outcomes are generally poor, even in well-resourced healthcare settings [30]. These outcomes are influenced by multiple factors, including the pre-arrest neurological status of the patient, the underlying etiology of the arrest, the quality and duration of CPR, the use of Extracorporeal CPR (E-CPR), and post-resuscitation measures particularly hemodynamic stability, and temperature control [26, 30–32]. In our setting, although advanced temperature modulation strategies such as targeted temperature management were not routinely available, low-cost neuroprotective measures were consistently implemented, including active fever prevention through temperature monitoring and treatment of hyperthermia, maintenance of adequate oxygenation, controlled ventilation, hemodynamic stabilization, glucose monitoring, and prompt seizure management.

Optimizing post–cardiac arrest neurological outcomes require not only high-quality in-hospital care but also robust public health and community-based interventions. There is strong evidence supporting the role of widespread awareness and training in bystander CPR, establishing efficient patient transportation systems and emergency hotline centers, developing well-structured referral networks, ensuring ambulances are properly equipped, and strengthening pre-hospital emergency care teams with highly skilled and experienced personnel [1, 21, 22]. In Afghanistan, however, access to basic life support (BLS) training for the general public is virtually non-existent, and pre-hospital ambulance and paramedical services are inadequate, even in urban areas. As a result, emergency response times are often prolonged, and most cardiac arrest patients are transported to hospitals in private vehicles by non-medical personnel, receiving little to no life support care during the critical pre-hospital phase. These gaps in the pre-hospital system likely contribute to poor outcomes and underscore the urgent need for healthcare system improvements, particularly in emergency response infrastructure and community-based resuscitation training programs.

In terms of cardiac arrest etiology, sepsis (60.2%) was the most frequently reported provisional diagnosis in our cohort, followed by lower respiratory tract infections (LRTIs). These findings are consistent with previous studies that primarily analyzed cardiac arrests in low- and middle-income countries (LMICs), where infectious diseases remain the predominant cause of cardiac arrest [20, 22, 27, 33].

Notably, we found sepsis to be an independent predictor of survival. This finding contrasts with previous studies, which have reported lower survival rates following sepsis-related cardiac arrest compared to other etiologies [20, 27, 30, 34]. Several factors may explain this discrepancy. First, our study revealed that several factors in the pre-arrest phase were significantly associated with survival. Having IV/IO access established prior to cardiac arrest was independently associated with more than threefold higher odds of survival. Additionally, monitoring at the time of arrest was significantly associated with improved survival outcomes. These findings suggest that these patients were already receiving timely and intensive medical management, including aggressive fluid resuscitation and or inotropes before the arrest occurred. Second, a substantial proportion of our study population experienced sepsis-related cardiac arrest, which may have influenced the overall survival outcomes. Recognizing the causes of pediatric cardiac arrest and their influence on survival is particularly challenging in low-income countries such as Afghanistan, where medical record systems and patient documentation are often inadequate. We therefore recommend that future prospective studies further investigate the etiology of pediatric cardiac arrest, especially sepsis-associated cardiac arrest and its survival outcomes in resource-limited settings of Afghanistan.

In our cohort, the initial cardiac rhythm was documented in 41.5% of cases overall. This finding is consistent with previous reports suggesting that in low-income settings, where most cardiac arrests are unwitnessed and unmonitored, the initial cardiac rhythm often remains unknown [30].

Our findings revealed that the most commonly recorded initial cardiac rhythms were non-shockable rhythms, with bradycardia observed in 41.5% of cases and asystole in 35.5% of cases. Shockable rhythms (VF/pVT) were rare, occurring in only 2% of cases. In line with previous studies, our findings suggest that most pediatric initial cardiac arrest rhythms are non-shockable. Shockable rhythms are often recorded as secondary rhythms during CPR or as initial rhythms in children with underlying cardiac disease or those who have undergone cardiac surgery [5, 20, 21]. In terms of survival outcomes, prior studies have found that shockable rhythms are associated with higher survival rates, particularly among adult patients. However, in our cohort, the initial cardiac rhythm was not significantly associated with survival outcomes [1, 5].

Our findings indicate that CPR duration is an independent predictor of survival, with shorter CPR duration being positively associated with achieving ROSC, survival to hospital discharge (STD), and favorable neurological outcomes. The median CPR duration in our cohort was 10 min, which aligns with previous studies [15, 20]. In our study, a CPR duration of ≤ 20 min was significantly associated with attaining ROSC in the ED, a duration of ≤ 15 min was positively associated with STD, and a CPR duration of ≤ 5 min was strongly associated with favorable neurological outcomes. In contrast, prolonged CPR beyond 20 min was an independent predictor of decreased survival. Specifically, CPR lasting more than 5 min was found to be one of the most important prognostic factors for unfavorable neurological outcomes in pediatric cardiac arrest cases in the ED.

Consistent with previous studies, we found that prolonged CPR in the ED is rarely effective and that the CPR duration should be considered when determining when to discontinue resuscitative efforts [18, 20–22]. Studies from low- and middle-income countries (LMICs) have consistently reported poor outcomes in children who underwent CPR for more than 15–20 min, with no survivors reported beyond 15 min of resuscitation [20, 22, 33, 35–37]. Söğütlü et al., in a pediatric in-hospital study that included pulseless arrest, respiratory arrest, and bradycardia cases, reported significantly longer CPR duration among non-survivors compared with survivors (median 45 vs. 15 min, *p* < 0.001), identifying prolonged CPR as an independent predictor of mortality. However, studies from high-income countries have reported survival even after 30 min of CPR [15]. In LMICs, decisions regarding the cessation of resuscitative efforts should not rely only on CPR duration. Other important factors, such as delayed presentation of critically ill patients, a high prevalence of malnutrition, and inadequate pre-hospital emergency care, should also be considered [20, 21].

It is well documented that the shorter the no-flow and low-flow phases of cardiac arrest, the better the outcomes. The no-flow phase should be shortened by closely monitoring high-risk patients to facilitate early recognition of cardiac arrest and the immediate initiation of resuscitation measures. During the low-flow phase, high-quality CPR optimizes coronary and cerebral perfusion pressure, ensuring adequate blood flow to vital organs. Longer duration of cardiac arrest leads to a sustained period of low cardiac output, resulting in decreased myocardial perfusion, reduced oxygen delivery, and subsequent organ injury. Consequently, patients requiring prolonged CPR are less likely to have improved survival outcomes [20, 21, 26].

The current study revealed that several factors related to the pre-arrest, intra-arrest, and post-arrest phases were positively associated with improved survival outcomes. These included having established IV/IO access prior to arrest, receiving oxygen therapy before arrest, being under monitoring at the time of arrest, a response time of ≤ 1 min, receiving ≤ 2 doses of epinephrine during CPR, administration of the first epinephrine dose within ≤ 2 min, and undergoing endotracheal intubation during CPR. These findings align with previous studies indicating that early administration of fewer doses of epinephrine is associated with better survival outcomes [18, 21]. A response time of ≤ 1 min and having established IV/IO access prior to arrest, which facilitates early epinephrine administration, were important predictors associated with better survival to hospital discharge with favorable neurological outcomes.

### Limitations of the study

This study has several limitations that should be considered when interpreting the findings. First, its retrospective design relied exclusively on existing clinical documentation. As a result, it was not possible to evaluate the quality of clinical care provided, an important factor that may have significantly influenced patient outcomes. Second, the study was conducted at a single tertiary care center, the French Medical Institute for Mothers and Children (FMIC) in Kabul, a specialized and well-equipped institution that predominantly serves urban populations. This limits the generalizability of the findings to other regions, particularly rural areas with different healthcare resources and patient profiles.

Third, the study focused exclusively on acute outcomes, including return of spontaneous circulation (ROSC), survival to hospital discharge, and neurological status at discharge. While these are the most important endpoints, they do not capture the full spectrum of recovery. Data on long-term survival and neurological outcomes were not collected, limiting the ability to assess full functional recovery over the long term. Fourth, some provisional diagnoses, particularly sepsis, may contribute to multiple downstream pathways leading to cardiac arrest. This overlap represents a limitation of our classification approach and should be considered when interpreting the diagnostic breakdown.

Fifth, some patients may experience recurrent cardiac arrest after ICU admission or later during the hospital course, which could affect overall survival outcomes. However, data on this were not recorded and therefore could not be analyzed. This represents a potential area for future research. Finally, the relatively small sample size and limited number of events may have contributed to wide confidence intervals for some predictors and increased the risk of model overfitting. These findings should therefore be interpreted with caution, and larger studies are needed to confirm these associations.

### Conclusion and recommendations

Our study found that the ROSC rate in the emergency department was 67% and the survival to hospital discharge rate was 30%, with 14.5% of patients discharged in a favorable neurological condition. Key factors associated with favorable post-cardiac arrest outcomes included monitoring at the time of arrest, response time ≤ 1 min, endotracheal intubation during CPR, administration of ≤ 2 doses of epinephrine during CPR, CPR duration ≤ 15 min, and post-resuscitation mechanical ventilation and vasopressors/inotropes therapy.

Based on these findings, we suggest the following recommendations for future researchers, healthcare professionals, and policymakers to improve cardiac arrest outcomes in Afghanistan:

1. Given the high mortality, poor outcomes, and lack of data on cardiac arrest in Afghanistan, further prospective and multicenter studies at the national level are needed to explore its causes, outcomes, and prognostic factors in both pediatric and adult patients, with an emphasis on out-of-hospital cardiac arrest.
2. Training and continuing education of medical assistants, physicians, and nurses through standardized courses in advanced life support (ALS) offered by a national medical organization. This includes Basic Life Support (BLS), Pediatric Advanced Life Support (PALS), and the Neonatal Resuscitation Program (NRP).
3. Public awareness campaigns on the importance of learning and administering bystander CPR for the general public, students, teachers, and staff in various settings, including physician offices, supermarkets, private and public organizations, schools, and universities. Resuscitation carts or kits containing essential resuscitation equipment and medications should be made available in these locations.
4. A centrally controlled ambulance service with trained personnel should be established to provide high-quality on-site and during-transfer care.
5. Standardized protocols for the telephone triage of critically ill patients, prehospital care, transportation, emergency department management, and in hospital care should be developed and implemented.
6. The integration of Extracorporeal CPR (E-CPR) and the use of Targeted Temperature Management (TTM) should be prioritized in clinical practice.
7. Immediate cardiac monitoring upon arrival should be ensured for at-risk patients to improve rhythm recognition and prompt defibrillation.

## Data Availability

The dataset supporting the conclusions of this study is available from the corresponding author upon reasonable request.

## Abbreviations

ALS: Advanced life support
AHA: American heart association
BLS: Basic life support
CA: Cardiac arrest
CPR: Cardiopulmonary resuscitation
DNR: Do not resuscitate
EDC: Education development center
HICs: High income countries
IV/IO: Intravenous or intraosseous
IQR: Interquartile range
LMICs: Low- and middle-income countries
OR: Odds ratio
pVT: Pulseless ventricular tachycardia
PEA: Pulseless electrical activity
PCPC: Pediatric cerebral performance category
ROSC: Return of spontaneous circulation
STD: Survival to discharge
STA: Survival to admission
TTM: Targeted temperature management
SVT: Supraventricular tachycardia
VF: Ventricular fibrillation
VT: Ventricular tachycardia
